# Linear and Fisher Separability of Random Points in the *d*-Dimensional Spherical Layer and Inside the *d*-Dimensional Cube

**DOI:** 10.3390/e22111281

**Published:** 2020-11-12

**Authors:** Sergey Sidorov, Nikolai Zolotykh

**Affiliations:** Institute of Information Technologies, Mathematics and Mechanics, Lobachevsky State University, 603950 Nizhni Novgorod, Russia; sergey.sidorov@itmm.unn.ru

**Keywords:** stochastic separation theorems, random points, 1-convex set, linear separability, Fisher separability, Fisher linear discriminant

## Abstract

Stochastic separation theorems play important roles in high-dimensional data analysis and machine learning. It turns out that in high dimensional space, any point of a random set of points can be separated from other points by a hyperplane with high probability, even if the number of points is exponential in terms of dimensions. This and similar facts can be used for constructing correctors for artificial intelligent systems, for determining the intrinsic dimensionality of data and for explaining various natural intelligence phenomena. In this paper, we refine the estimations for the number of points and for the probability in stochastic separation theorems, thereby strengthening some results obtained earlier. We propose the boundaries for linear and Fisher separability, when the points are drawn randomly, independently and uniformly from a *d*-dimensional spherical layer and from the cube. These results allow us to better outline the applicability limits of the stochastic separation theorems in applications.

## 1. Introduction

It is generally accepted that the modern information world is the world of big data. However, some of the implications of the advent of the big data era remain poorly understood. In his “millennium lecture”, D. L. Donoho [1] described the post-classical world in which the number of features *d* is much greater than the sample size *n*: d≫n. It turns out that many phenomena of the post-classical world are already observed if d≫logn, or, more precisely, when ID≫logn, where ID is the intrinsic dimensionality of the data [2]. Classical methods of data analysis and machine learning become of little use in such a situation, because usually they require huge amounts of data. Such an unlimited appetite of classical approaches for data is usually considered as a phenomenon of the “curse of dimensionality”. However, the properties ID≫n or ID≫logn themselves are neither a curse nor a blessing, and can be beneficial.

One of the “post-classical” phenomena is stochastic separability [3,4,5]. If the dimensionality of data is high, then under broad assumptions any sample of the data set can be separated from the rest by a hyperplane (or even Fisher discriminant—as a special case) with a probability close to 1 even the number of samples is exponential in terms of dimensions. Thus, high-dimensional datasets exhibit fairly simple geometric properties.

Recently, stochastic separation theorems have been widely used in machine learning for constructing correctors and ensembles of correctors of artificial intelligence systems [6,7], for determining the intrinsic dimensionality of data sets [8,9], for explaining various natural intelligence phenomena, such as grandmother’s neuron [10,11].

In its usual form a stochastic separation theorem is formulated as follows. A random *n*-element set in $R^{d}$ is linearly separable with probability p>1−ϑ, if $n<ae^{bd}$. The exact form of the exponential function depends on the probability distribution that determines how the random set is drawn, and on the constant ϑ (0<ϑ<1). In particular, uniform distributions with different support are considered in [5,12,13,14]. Wider classes of distributions (including non-i.i.d.) are considered in [7]. Roughly speaking, these classes consist of distributions without sharp peaks in sets with exponentially small volume. Estimates for product distributions in the cube and the standard normal distribution are obtained in [15]. General stochastic separation theorems with optimal bounds for important classes of distributions (log-concave distribution, their convex combinations and product distributions) are proposed in [2].

We note that there are many algorithms for constructing a functional separating a point from all other points in a data set (Fisher linear discriminant, linear programming algorithm, support vector machine, Rosenblatt perceptron, etc.). Among all these methods the computationally cheapest is Fisher discriminant analysis [6]. Other advantages of the Fisher discriminant analysis are its simplicity and the robustness.

The papers [5,6,7,12] deal with only Fisher separability, whereas [13,14] considered a (more general) linear separability. A comparison of the estimations for linear and Fisher separability allows us to clarify the applicability boundary of these methods, namely, to answer the question of what *d* and *n* are sufficient in order to use only Fisher separability and so that there is no need to search a more sophisticated linear discriminant.

In [13,14], there were obtained estimates for the cardinality of the set of points that guarantee its linear separability when the points are drawn randomly, independently and uniformly from a *d*-dimensional spherical layer and from the unit cube. These results give more accurate estimates than the bounds obtained in [5,12] for Fisher separability.

Our interest in the study of the linear separability in spherical layers is explained, among other reasons, by the possibility of applying our results to determining the intrinsic dimension of data. After applying PCA to the data points for the selection of the major components and subsequent whitening we can map them to a spherical layer of a given thickness. If the intrinsic dimensionality of the initial set of *n* points is ID, then we expect that the separability properties of the resulting set of points are similar to the properties of uniformly distributed *n* points in dimension *d*. In particular, we can use the theoretical estimates for the separation probability to estimate ID (cf. [8,9]).

Here we give even more precise estimations for the number of points in the spherical layer to guarantee their linear separability. We also consider the case of linear separability of random points inside a cube in more detail than it was done in [13]. In particular, we give estimates for the probability of separability of one point. We also report results of computational experiments comparing the theoretical estimations for the probability of the linear and Fisher separabilities with the corresponding experimental frequencies and discuss them.

## 2. Definitions

A point $X\in R^{d}$ is linearly separable from a set $M\subset R^{d}$ if there exists a hyperplane separated *X* from *M*; i.e., there exists $A_{X}\in R^{d}$ such that $\left(A_{X},X\right)>\left(A_{X},Y\right)$ for all Y∈M.

A point $X\in R^{d}$ is Fisher separable from the set $M\subset R^{d}$ if (X,Y)<(X,X) for all Y∈M [6,7].

A set of points $\left\{X_{1},\ldots ,X_{n}\right\}\subset R^{d}$ is called linearly separable [5] or 1-convex [3] if any point $X_{i}$ is linearly separable from all other points in the set, or in other words, the set of vertices of their convex hull, $\mathrm{conv}(X_{1},\ldots ,X_{n})$, coincides with $\left\{X_{1},\ldots ,X_{n}\right\}$. The set $\left\{X_{1},\ldots ,X_{n}\right\}$ is called Fisher separable if $\left(X_{i},X_{j}\right)<\left(X_{i},X_{i}\right)$ for all *i*, *j*, such that i≠j [6,7].

Fisher separability implies linear separability but not vice versa (even if the set is centered and normalized to unit variance). Thus, if $M\subset R^{d}$ is a random set of points from a certain probability distribution, then the probability that *M* is linearly separable is not less than the probability that *M* is Fisher separable.

Denote by $B_{d}=\{X\in R^{d}:\;∥X∥\leq 1\}$ the *d*-dimensional unit ball centered at the origin (∥X∥ means Euclidean norm), $rB_{d}$ is the *d*-dimensional ball of radius r<1 centered at the origin and $Q_{d}={\left[0,1\right]}^{d}$ is the *d*-dimensional unit cube.

Let $M_{n}=\left\{X_{1},\ldots ,X_{n}\right\}$ be the set of points chosen randomly, independently, according to the uniform distribution on the (1−r)-thick spherical layer $B_{d}\backslash rB_{d}$, i.e., on the unit ball with spherical cavity of radius *r*. Denote by $P^{\circ }\left(d,r,n\right)$ the probability that $M_{n}$ is linearly separable, and by $P^{\circ F}\left(d,r,n\right)$ the probability that $M_{n}$ is Fisher separable. Denote by $P_{1}^{\circ }\left(d,r,n\right)$ the probability that a random point chosen according to the uniform distribution on $B_{d}\backslash rB_{d}$ is separable from $M_{n}$, and by $P_{1}^{\circ F}\left(d,r,n\right)$ the probability that a random point is Fisher separable from $M_{n}$.

Now let $M_{n}=\left\{X_{1},\ldots ,X_{n}\right\}$ be the set of points chosen randomly, independently, according to the uniform distribution on the cube $Q_{d}$. Let $P^{\;□}\left(d,n\right)$ and $P^{\;□F}\left(d,n\right)$ denote the probabilities that $M_{n}$ is linearly separable and Fisher separable, respectively. Let $P_{1}^{\;□}\left(d,n\right)$ and $P_{1}^{\;□F}\left(d,n\right)$ denote the probabilities that a random point chosen according to the uniform distribution on $Q_{d}$ is separable and Fisher separable from $M_{n}$, respectively.

## 3. Previous Results

### 3.1. Random Points in a Spherical Layer

In [5] it was shown (among other results) that for all *r*, ϑ, *n* and *d*, where 0<r<1, 0<ϑ<1, d∈N, if

(1) $$n<{\left(\frac{r}{\sqrt{1-r^{2}}}\right)}^{d}\left(\sqrt{1+\frac{2\vartheta {\left(1-r^{2}\right)}^{d/2}}{r^{2d}}}-1\right),$$

then *n* points chosen randomly, independently, according to the uniform distribution on $B_{d}\backslash rB_{d}$ are Fisher separable with a probability greater than 1−ϑ, i.e., $P^{\circ F}\left(d,r,n\right)>1-\vartheta$.

The following statements concerning the Fisher separability of random points in the spherical layer are proved in [12].

- For all *r*, where 0<r<1, and for any d∈N
  (2) $$P_{1}^{\circ F}\left(d,r,n\right)>\left(1-r^{d}\right){\left(1-\frac{{\left(1-r^{2}\right)}^{d/2}}{2}\right)}^{n}.$$
- For all *r*, ϑ, where 0<r<1, 0<ϑ<1, and for sufficiently large *d*, if
  (3) $$n<\frac{\vartheta }{{\left(1-r^{2}\right)}^{d/2}},$$
  then $P_{1}^{\circ F}\left(d,r,n\right)>1-\vartheta$.
- For all *r*, where 0<r<1, and for any d∈N
  (4) $$P^{\circ F}\left(d,r,n\right)>{\left[\left(1-r^{d}\right)\left(1-\left(n-1\right)\frac{{\left(1-r^{2}\right)}^{d/2}}{2}\right)\right]}^{n}.$$
- For all *r*, ϑ, where 0<r<1, 0<ϑ<1 and for sufficiently large *d*, if
  (5) $$n<\frac{\sqrt{\vartheta }}{{\left(1-r^{2}\right)}^{d/4}},$$
  then $P^{\circ F}\left(d,r,n\right)>1-\vartheta$.

The authors of [5,12] formulate their results for linearly separable sets of points, but in fact in the proofs they used that the sets are only Fisher separable.

Note that all estimates (1)–(5) require 0<r<1 with strong inequality. This means that they are inapplicable for (maybe the most interesting) case r=0, i.e., for the unit ball with no cavities.

A reviewer of the original version of the article drew our attention that for r=0 better results are obtained in [6,15]. Specifically,

(6) $$P_{1}^{\circ F}\left(d,0,n\right)\geq 1-\frac{n}{2^{d+1}},$$

(7) $$P^{\circ F}\left(d,0,n\right)\geq 1-\frac{n(n-1)}{2^{d+1}}>1-\frac{n^{2}}{2^{d+1}},$$

and $P_{1}^{\circ F}\left(d,0,n\right)>1-\vartheta$ provided that $n<\vartheta \cdot 2^{d+1}$. See details in Section 4.4.

The both estimates (1) and (5) are exponentially dependent on *d* for fixed *r*, ϑ and the estimate (1) is weaker than (5).

The following results concerning the linear separability of random points in the spherical layer were obtained in [14]:

- For all *r*, where 0≤r<1, and for any d∈N
  (8) $$P_{1}^{\circ }\left(d,r,n\right)>1-\frac{n}{2^{d}}.$$
- For all *r*, ϑ, where 0≤r<1, 0<ϑ<1, and for any d∈N, if
  (9) $$n<\vartheta 2^{d},$$
  then $P_{1}^{\circ }\left(d,r,n\right)>1-\vartheta .$
- For all *r*, where 0≤r<1, and for any d∈N
  (10) $$P^{\circ }\left(d,r,n\right)>1-\frac{n(n-1)}{2^{d}}.$$
- For all *r*, ϑ, where 0≤r<1, 0<ϑ<1, and for any *d*, if
  (11) $$n<\sqrt{\vartheta 2^{d}},$$
  then $P^{\circ }\left(d,r,n\right)>1-\vartheta .$

We note that the bounds (8)–(11) do not depend on *r*. We remove this drawback in this paper, giving more accurate estimates (see Theorems 1 and 3 and Corollaries 1 and 2).

### 3.2. Random Points Inside a Cube

In [5], a product distribution in the $Q_{d}$ is considered. Let the coordinates of a random point $X=\left(x_{1},\ldots ,x_{d}\right)\in Q_{d}$ be independent random variables with variances $\sigma_{i}^{2}>\sigma_{0}^{2}>0$ (i=1,…,d). In [5], it is shown that for all ϑ and *n*, where 0<ϑ<1, if

(12) $$n<\sqrt{\frac{\vartheta e^{0.5d\sigma_{0}^{4}}}{3}},$$

then $M_{n}$ is Fisher separable with a probability greater than 1−ϑ. As above, the authors of [5] formulate their result for the linearly separable case, but in fact they used only the Fisher separability.

If all random variables $x_{1},\ldots ,x_{d}$ have the uniform distribution on the segment [0,1] then $\sigma_{0}^{2}=\frac{1}{12}.$ Thus, the inequality (12) takes the form

(13) $$n<\sqrt{\frac{\vartheta e^{d/288}}{3}}.$$

We obtain that if *n* satisfies (13), then $P^{\;□F}\left(d,n\right)>1-\vartheta$.

In [13], it was shown that if we want to guarantee only the linear separability, then the bound (13) can be increased. Namely, if

$$n<\sqrt{\frac{\vartheta c^{d}}{d+1}},\;c=1.18858,$$

then $P^{\;□}\left(d,n\right)>1-\vartheta$. Here we give related estimates including ones for the linear separability of one point (see Theorems 5 and 6 and Corollary 3).

We note that better (and in fact asymptotically optimal) estimates for the Fisher separability in the unit cube are derived in [15]. The papers [13,15] were submitted to the same conference, so these results were derived in parallel and independently. Corollary 7 in [15] states that *n* points are Fisher separable with probability greater than 1−ϑ provided only that $n<\sqrt{\vartheta }e^{\gamma d}$ for γ=0.23319… See details in Section 5.

## 4. Random Points in a Spherical Layer

### 4.1. The Separability of One Point

The theorem below gives the probability of the linear separability of a random point from a random *n*-element set $M_{n}=\left\{X_{1},\ldots ,X_{n}\right\}$ in $B_{d}\backslash rB_{d}.$ The proof develops an approach borrowed from [3,16].

The regularized incomplete beta function is defined as $I_{x}\left(a,b\right)=\frac{B(x;a,b)}{B(a,b)}$, where

$$B\left(a,b\right)=\int_{0}^{1}t^{a-1}{\left(1-t\right)}^{b-1}dt,\;B\left(x;a,b\right)=\int_{0}^{x}t^{a-1}{\left(1-t\right)}^{b-1}dt$$

are beta function and incomplete beta function, respectively (see [17]).

**Theorem** **1.** *Let 0≤r<1, $\alpha =4r^{2}\left(1-r^{2}\right),$$\beta =1-r^{2},$d∈N. Then*

*(1)*  *for $0\leq r\leq \frac{1}{\sqrt{2}}$*
  (14) $$P_{1}^{\circ }\left(d,r,n\right)>1-n\cdot \frac{1-0.5\left(I_{\alpha }\left(\frac{d+1}{2},\frac{1}{2}\right)+{\left(2r\right)}^{d}\cdot I_{\beta }\left(\frac{d+1}{2},\frac{1}{2}\right)\right)}{2^{d}\left(1-r^{d}\right)};$$
*(2)*  *for $\frac{1}{\sqrt{2}}\leq r<1$*
  (15) $$P_{1}^{\circ }\left(d,r,n\right)>1-n\cdot \frac{0.5\left(I_{\alpha }\left(\frac{d+1}{2},\frac{1}{2}\right)-{\left(2r\right)}^{d}\cdot I_{\beta }\left(\frac{d+1}{2},\frac{1}{2}\right)\right)}{2^{d}\left(1-r^{d}\right)}.$$

**Proof.**  A random point *Y* is linearly separable from $M_{n}=\left\{X_{1},\ldots ,X_{n}\right\}$ if and only if $Y\notin \mathrm{conv}(M_{n}).$ Denote this event by C. Thus, $P_{1}^{\circ }\left(d,r,n\right)=P\left(C\right).$ Let us find the upper bound for the probability of the event $\bar{C}.$ This event means that the point *Y* belongs to the convex hull of $M_{n}.$ Since the points in $M_{n}$ have the uniform distribution, then the probability of $\bar{C}$ is

$$P\left(\bar{C}\right)=\frac{\mathrm{Vol}(\mathrm{conv}\left(M_{n}\right)\backslash (\mathrm{conv}\left(M_{n}\right)\cap rB_{d}))}{\mathrm{Vol}\left(B_{d}\right)-\mathrm{Vol}\left(rB_{d}\right)}.$$

**Figure 1:**
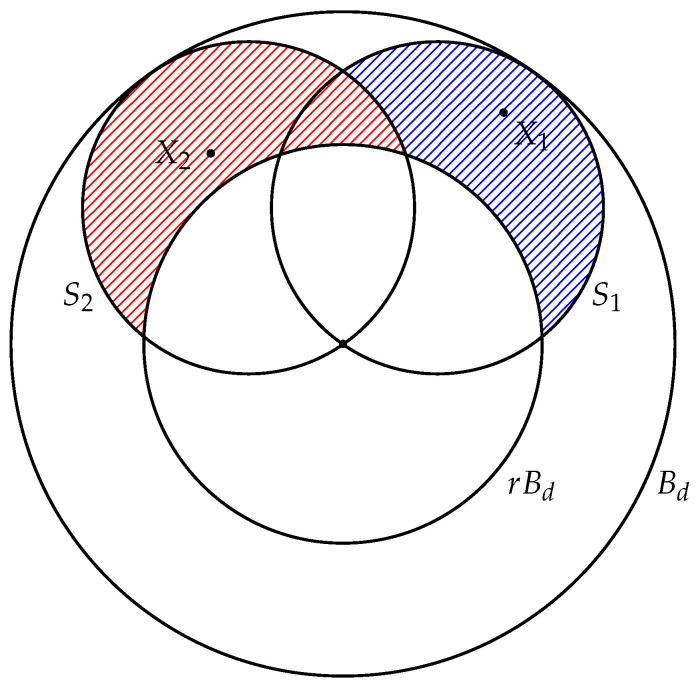
Illustration to the proof of Theorem 1.

First, estimate the numerator of this fraction. We denote by $S_{i}$ the ball with center at the origin, with the diameter 1, and the point $X_{i}$ lies on this diameter (see Figure 1). Then

$$\mathrm{conv}\left(M_{n}\right)\backslash \left(\mathrm{conv}\left(M_{n}\right)\cap rB_{d}\right)\subseteq \overset{n}{\underset{i=1}{⋃}}\left(S_{i}\backslash \left(S_{i}\cap rB_{d}\right)\right)=W$$

and

$$\mathrm{Vol}(\mathrm{conv}\left(M_{n}\right)\backslash (\mathrm{conv}\left(M_{n}\right)\cap rB_{d}))\leq \mathrm{Vol}\left(W\right)\leq \sum_{i=1}^{n}\mathrm{Vol}\left(S_{i}\backslash \left(S_{i}\cap rB_{d}\right)\right)$$

$$=\sum_{i=1}^{n}\left(\mathrm{Vol}\left(S_{i}\right)-\mathrm{Vol}\left(S_{i}\cap rB_{d}\right)\right)=n\left(\mathrm{Vol}\left(S_{1}\right)-\mathrm{Vol}\left(S_{1}\cap rB_{d}\right)\right)$$

$$=n\left(\gamma_{d}{\left(\frac{1}{2}\right)}^{d}-\mathrm{Vol}\left(S_{1}\cap rB_{d}\right)\right),$$

where $\gamma_{d}$ is the volume of a ball of radius 1. Hence

$$P\left(\bar{C}\right)\leq \frac{n\left(\gamma_{d}{\left(\frac{1}{2}\right)}^{d}-\mathrm{Vol}\left(S_{1}\cap rB_{d}\right)\right)}{\gamma_{d}\left(1-r^{d}\right)}.$$

Now find $\mathrm{Vol}(S_{1}\cap rB_{d}).$ It is obvious that $\mathrm{Vol}(S_{1}\cap rB_{d})$ is equal to the sum of the volumes of two spherical caps. We denote by Cap(R,H) the volume of a spherical cap of height *H* of a ball of radius R. It is known [18] that

$$\mathrm{Cap}\left(R,H\right)=\frac{1}{2}\gamma_{d}R^{d}I_{\left(2RH-H^{2}\right)/R^{2}}\left(\frac{d+1}{2},\frac{1}{2}\right)$$

if 0≤H≤R.

**Figure 2:**
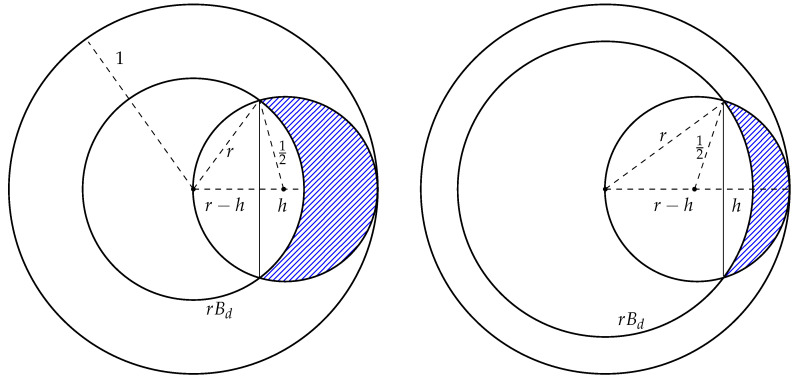
Illustration to the proof of Theorem 1: case 1 (**left**); case 2 (**right**).

Consider two cases: $0\leq r\leq \frac{1}{\sqrt{2}}$ and $\frac{1}{\sqrt{2}}\leq r<1$ (see Figure 2)

**Case 1** If $0\leq r\leq \frac{1}{\sqrt{2}}$, then the centers of the balls $S_{1},S_{2},\ldots ,S_{n}$ are inside of the spherical caps of height *h* of the ball $rB_{d}$ (see the left picture on Figure 2). Therefore, the following equalities are true:

$$r^{2}-{\left(r-h\right)}^{2}={\left(\frac{1}{2}\right)}^{2}-{\left(r-h-\frac{1}{2}\right)}^{2},$$

$$r^{2}-{\left(r-h\right)}^{2}=-{\left(r-h\right)}^{2}+\left(r-h\right),$$

$$h=r-r^{2},$$

$$V_{1}=\mathrm{Cap}\left(\frac{1}{2},r-h\right)=\mathrm{Cap}\left(\frac{1}{2},r^{2}\right),\;V_{2}=\mathrm{Cap}\left(r,h\right)=\mathrm{Cap}\left(r,r-r^{2}\right).$$

If $R=\frac{1}{2},$ $H=r^{2}$, then $\left(2RH-H^{2}\right)/R^{2}=4r^{2}\left(1-r^{2}\right)=\alpha$, hence

$$V_{1}=\frac{1}{2}\gamma_{d}{\left(\frac{1}{2}\right)}^{d}I_{\alpha }\left(\frac{d+1}{2},\frac{1}{2}\right).$$

If R=r, $H=r-r^{2}$, then $\left(2RH-H^{2}\right)/R^{2}=2H/R-{\left(H/R\right)}^{2}=2\left(1-r\right)-{\left(1-r\right)}^{2}=1-r^{2}=\beta$, hence

$$V_{2}=\frac{1}{2}\gamma_{d}r^{d}I_{\beta }\left(\frac{d+1}{2},\frac{1}{2}\right).$$

Thus,

$$\mathrm{Vol}\left(S_{1}\cap rB_{d}\right)=V_{1}+V_{2}=\gamma_{d}\left(\frac{1}{2}{\left(\frac{1}{2}\right)}^{d}I_{\alpha }\left(\frac{d+1}{2},\frac{1}{2}\right)+\frac{1}{2}r^{d}I_{\beta }\left(\frac{d+1}{2},\frac{1}{2}\right)\right).$$

Hence

$$P\left(C\right)=1-P\left(\bar{C}\right)\geq 1-\frac{n\left(\gamma_{d}{\left(\frac{1}{2}\right)}^{d}-\mathrm{Vol}\left(S_{1}\cap rB_{d}\right)\right)}{\gamma_{d}\left(1-r^{d}\right)}$$

$$=1-n\cdot \frac{1-0.5\left(I_{\alpha }\left(\frac{d+1}{2},\frac{1}{2}\right)+{\left(2r\right)}^{d}\cdot I_{\beta }\left(\frac{d+1}{2},\frac{1}{2}\right)\right)}{2^{d}\left(1-r^{d}\right)}.$$

**Case 2** If $\frac{1}{\sqrt{2}}\leq r<1$, then the centers of the balls $S_{1},S_{2},\ldots ,S_{n}$ are outside of the spherical caps of height *h* of the ball $rB_{d}$ (see the right picture on Figure 2). Therefore, the following equalities are true:

$$r^{2}-{\left(r-h\right)}^{2}={\left(\frac{1}{2}\right)}^{2}-{\left(r-h-\frac{1}{2}\right)}^{2},$$

$$r^{2}-{\left(r-h\right)}^{2}=-{\left(r-h\right)}^{2}+\left(r-h\right),$$

$$h=r-r^{2},$$

$$V_{1}=\mathrm{Vol}\left(\frac{1}{2}B_{d}\right)-\mathrm{Cap}\left(\frac{1}{2},1-\left(r-h\right)\right)=\mathrm{Vol}\left(\frac{1}{2}B_{d}\right)-\mathrm{Cap}\left(\frac{1}{2},1-r^{2}\right).$$

If $R=\frac{1}{2},$ $H=1-r^{2}$, then $\left(2RH-H^{2}\right)/R^{2}=4r^{2}\left(1-r^{2}\right)$; hence,

$$V_{1}=\gamma_{d}{\left(\frac{1}{2}\right)}^{d}-\frac{1}{2}\gamma_{d}{\left(\frac{1}{2}\right)}^{d}I_{\alpha }\left(\frac{d+1}{2},\frac{1}{2}\right),$$

where $\alpha =4r^{2}\left(1-r^{2}\right),$

$$V_{2}=\mathrm{Cap}\left(r,h\right)=\mathrm{Cap}\left(r,r-r^{2}\right)=\frac{1}{2}\gamma_{d}r^{d}I_{\beta }\left(\frac{d+1}{2},\frac{1}{2}\right),$$

where $\beta =1-r^{2}.$ Thus,

$$\mathrm{Vol}\left(S_{1}\cap rB_{d}\right)=V_{1}+V_{2}=\gamma_{d}\left({\left(\frac{1}{2}\right)}^{d}-\frac{1}{2}{\left(\frac{1}{2}\right)}^{d}I_{\alpha }\left(\frac{d+1}{2},\frac{1}{2}\right)+\frac{1}{2}r^{d}I_{\beta }\left(\frac{d+1}{2},\frac{1}{2}\right)\right).$$

Hence

$$P\left(C\right)=1-P\left(\bar{C}\right)\geq 1-\frac{n\left(\gamma_{d}{\left(\frac{1}{2}\right)}^{d}-\mathrm{Vol}\left(S_{1}\cap rB_{d}\right)\right)}{\gamma_{d}\left(1-r^{d}\right)}=1-n\cdot \frac{0.5\left(I_{\alpha }\left(\frac{d+1}{2},\frac{1}{2}\right)-{\left(2r\right)}^{d}\cdot I_{\beta }\left(\frac{d+1}{2},\frac{1}{2}\right)\right)}{2^{d}\left(1-r^{d}\right)}.$$

**Figure 3:**
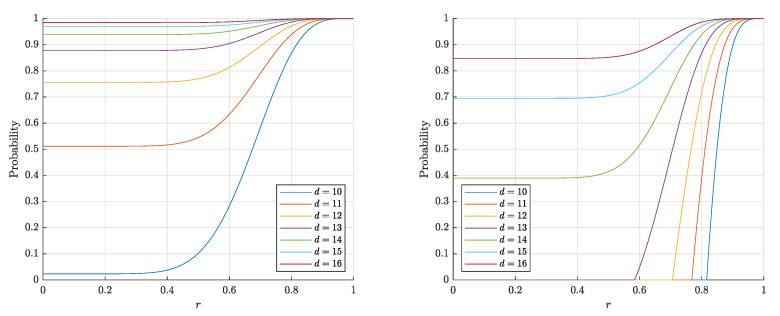
The graphs of the right-hand sides of the estimates (14), (15) for the probability $P_{1}^{\circ }\left(d,r,n\right)$ that a random point is linear and separable from a set of n=1000 (**left**) and n= 10,000 (**right**) random points in the layer $B_{d}\backslash rB_{d}$.

**Figure 4:**
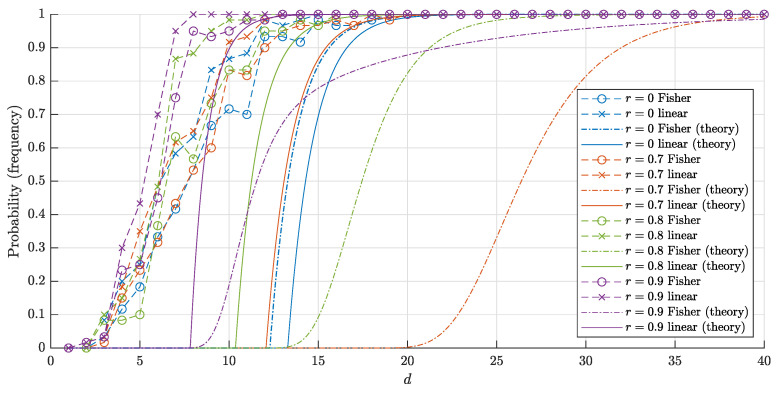
The graphs of the estimates for the probabilities $P_{1}^{\circ }\left(d,r,n\right)$ ($P_{1}^{\circ F}\left(d,r,n\right)$) that a random point is linearly (and respectively, Fisher) separable from a set of n= 10,000 random points in the layer $B_{d}\backslash rB_{d}$. The solid lines correspond to the theoretical bounds (14) and (15) for the linear separability. The dash-dotted lines represent the theoretical bounds (2) and (6) for the Fisher separability. The crosses (circles) correspond to the empirical frequencies for linear (and respectively Fisher) separability obtained in 60 trials for each dimension *d*.

**Figure 5:**
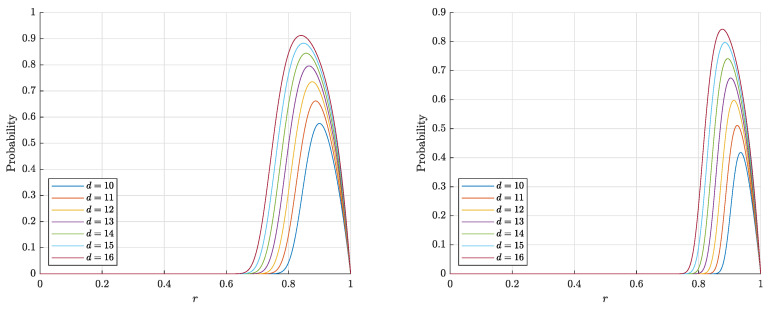
The graphs of the right-hand side of the estimate (3) for the probability $P_{1}^{\circ F}\left(d,r,n\right)$ that a random point is Fisher separable from a set of n=1000 (**left**) and n= 10,000 (**right**) random points in the layer $B_{d}\backslash rB_{d}$.

The estimates (14) and (15) for $P_{1}^{\circ }\left(d,r,n\right)$ are monotonically increasing in both *d* and *r* and decreasing in *n*, which corresponds to the behavior of the probability $P_{1}^{\circ }\left(d,r,n\right)$ itself (see Figure 3 and Figure 4). On the contrary, the estimate (3) for the probability $P_{1}^{\circ F}\left(d,r,n\right)$ is nonmonotonic in *r* (see Figure 5).

Note that the estimates (14), (15) obtained in Theorem 1 are quite accurate (in the sense that they are close to empirical values), as is illustrated with Figure 4. The experiment also shows that the probabilities $P_{1}^{\circ }\left(d,r,n\right)$ and $P_{1}^{\circ F}\left(d,r,n\right)$ (more precisely, the corresponding frequencies) are quite close to each other, but there is a certain gap between them.

The following corollary gives an estimate for the number of points *n* guaranteeing the linear separability of a random point from a random *n*-element set $M_{n}$ in $B_{d}\backslash rB_{d}$ with probability close to 1.

**Corollary** **1.** *Let 0<ϑ<1,$\alpha =4r^{2}\left(1-r^{2}\right),$$\beta =1-r^{2},$d∈N. If*

*(1)*  $$n<N_{1}\left(d,r,\vartheta \right)=\frac{\vartheta 2^{d}\left(1-r^{d}\right)}{1-0.5\left(I_{\alpha }\left(\frac{d+1}{2},\frac{1}{2}\right)+{\left(2r\right)}^{d}\cdot I_{\beta }\left(\frac{d+1}{2},\frac{1}{2}\right)\right)},\;0\leq r\leq \frac{1}{\sqrt{2}}$$
  *or*
*(2)*  $$n<N_{2}\left(d,r,\vartheta \right)=\frac{\vartheta 2^{d}\left(1-r^{d}\right)}{0.5\left(I_{\alpha }\left(\frac{d+1}{2},\frac{1}{2}\right)-{\left(2r\right)}^{d}\cdot I_{\beta }\left(\frac{d+1}{2},\frac{1}{2}\right)\right)},\;\frac{1}{\sqrt{2}}\leq r<1,$$

*then $P_{1}^{\circ }\left(d,r,n\right)>1-\vartheta .$*

The theorem below establishes asymptotic estimates.

**Theorem** **2.**

*(1)*  *If $0\leq r<\frac{1}{\sqrt{2}}$ then*
  $$N_{1}\left(d,r,\vartheta \right)∼\vartheta 2^{d}.$$
*(2)*  *If $r=\frac{1}{\sqrt{2}}$ then*
  $$N_{1}\left(d,r,\vartheta \right)=N_{2}\left(d,r,\vartheta \right)∼\vartheta 2^{d+1}.$$
*(3)*  *If $\frac{1}{\sqrt{2}}<r<1$ then*
  $$N_{2}\left(d,r,\vartheta \right)∼\vartheta \sqrt{2\pi }\cdot \frac{r(2r^{2}-1)}{\sqrt{1-r^{2}}}\cdot \sqrt{d+1}\cdot {\left(\frac{1}{r\sqrt{1-r^{2}}}\right)}^{d}.$$

**Proof.**  The paper [19] gives the following asymptotic expansion for the incomplete beta function

$$B\left(x;a,b\right)∼\frac{x^{a}}{a}\sum_{k=0}^{\infty }\frac{f_{k}\left(b,x\right)}{a^{k}}\;\mathrm{for}\;0\leq x<1,\;a\to \infty$$

and

$$f_{k}\left(b,x\right)=\frac{d^{k}}{dw^{k}}{\left[{\left(1-xe^{-w}\right)}^{b-1}\right]}_{w=0}.$$

Since $f_{0}\left(b,x\right)={\left(1-x\right)}^{b-1}$ then

$$B\left(x;a,b\right)∼\frac{x^{a}}{a}{\left(1-x\right)}^{b-1}+\frac{x^{a}}{a}\sum_{k=1}^{\infty }\frac{f_{k}\left(b,x\right)}{a^{k}}∼\frac{x^{a}}{a}{\left(1-x\right)}^{b-1}\;\mathrm{for}\;b,\;x\;\mathrm{fixed},\;a\to \infty .$$

Since $B\left(a,b\right)∼\frac{\Gamma (b)}{a^{b}}$ for *b* fixed and a→∞, then

$$I_{x}\left(a,b\right)=\frac{B(x;a,b)}{B(a,b)}∼\frac{x^{a}{\left(1-x\right)}^{b-1}}{a^{1-b}\Gamma \left(b\right)}$$

for b,x fixed and a→∞.

We have $x=\alpha =4r^{2}\left(1-r^{2}\right)$ or $x=\beta =1-r^{2}$ and $a=\frac{d+1}{2},$ $b=\frac{1}{2}$; hence,

$$I_{\alpha }\left(\frac{d+1}{2},\frac{1}{2}\right)∼\frac{\sqrt{2}\alpha^{\frac{d+1}{2}}}{\sqrt{\pi }\sqrt{d+1}\sqrt{1-4r^{2}+4r^{4}}}=\sqrt{\frac{2}{\pi }}\cdot \frac{1}{|1-2r^{2}|}\cdot \frac{\alpha^{\frac{d+1}{2}}}{\sqrt{d+1}},$$

$${\left(2r\right)}^{d}I_{\beta }\left(\frac{d+1}{2},\frac{1}{2}\right)∼\frac{{\left(2r\right)}^{d}\sqrt{2}{\left(\sqrt{1-r^{2}}\right)}^{d+1}}{r\sqrt{\pi }\sqrt{d+1}}=\sqrt{\frac{2}{\pi }}\cdot \frac{1}{2r^{2}}\cdot \frac{\alpha^{\frac{d+1}{2}}}{\sqrt{d+1}}.$$

If r=0, then α=0, β=1; hence, $N_{1}\left(d,r,\vartheta \right)∼\vartheta 2^{d}.$

If $0<r<\frac{1}{\sqrt{2}}$, then 0<α<1; hence,

$$I_{\alpha }\left(\frac{d+1}{2},\frac{1}{2}\right)+{\left(2r\right)}^{d}I_{\beta }\left(\frac{d+1}{2},\frac{1}{2}\right)∼0$$

and

$$N_{1}\left(d,r,\vartheta \right)∼\vartheta 2^{d}.$$

If $r=\frac{1}{\sqrt{2}}$, then α=1, $\beta =\frac{1}{2}$; hence,

$$N_{1}\left(d,r,\vartheta \right)=N_{2}\left(d,r,\vartheta \right)∼\frac{\vartheta 2^{d}\left(1-r^{d}\right)}{0.5\left(1-\sqrt{\frac{2}{\pi }}\cdot \frac{1}{\sqrt{d+1}}\right)}∼\vartheta 2^{d+1}.$$

If $\frac{1}{\sqrt{2}}<r<1$, then 0<α<1; hence,

$$I_{\alpha }\left(\frac{d+1}{2},\frac{1}{2}\right)-{\left(2r\right)}^{d}I_{\beta }\left(\frac{d+1}{2},\frac{1}{2}\right)∼\sqrt{\frac{2}{\pi }}\cdot \frac{1}{2r^{2}\left(2r^{2}-1\right)}\cdot \frac{\alpha^{\frac{d+1}{2}}}{\sqrt{d+1}}=\sqrt{\frac{2}{\pi }}\cdot \frac{\sqrt{1-r^{2}}}{r(2r^{2}-1)}\cdot \frac{2^{d}{\left(r\sqrt{1-r^{2}}\right)}^{d}}{\sqrt{d+1}}$$

and

$$N_{2}\left(d,r,\vartheta \right)=\frac{\vartheta 2^{d}\left(1-r^{d}\right)}{0.5\left(I_{\alpha }\left(\frac{d+1}{2},\frac{1}{2}\right)-{\left(2r\right)}^{d}\cdot I_{\beta }\left(\frac{d+1}{2},\frac{1}{2}\right)\right)}∼\frac{\vartheta 2^{d}}{0.5\sqrt{\frac{2}{\pi }}\cdot \frac{\sqrt{1-r^{2}}}{r(2r^{2}-1)}\cdot \frac{2^{d}{\left(r\sqrt{1-r^{2}}\right)}^{d}}{\sqrt{d+1}}}$$

$$=\vartheta \sqrt{2\pi }\cdot \frac{r(2r^{2}-1)}{\sqrt{1-r^{2}}}\cdot \sqrt{d+1}\cdot {\left(\frac{1}{r\sqrt{1-r^{2}}}\right)}^{d}.$$

□

### 4.2. Separability of a Set of Points

The theorem below gives the probability of the linear separability of a random *n*-element set $M_{n}$ in $B_{d}\backslash rB_{d}$.

**Theorem** **3.** *Let 0≤r<1, $\alpha =4r^{2}\left(1-r^{2}\right),$$\beta =1-r^{2}$ and d,n∈N. Then*

*(1)*  *for $0\leq r\leq \frac{1}{\sqrt{2}}$*
  (16) $$P^{\circ }\left(d,r,n\right)>1-n\left(n-1\right)\cdot \frac{1-0.5\left(I_{\alpha }\left(\frac{d+1}{2},\frac{1}{2}\right)+{\left(2r\right)}^{d}\cdot I_{\beta }\left(\frac{d+1}{2},\frac{1}{2}\right)\right)}{2^{d}\left(1-r^{d}\right)};$$
*(2)*  *for $\frac{1}{\sqrt{2}}\leq r<1$*
  (17) $$P^{\circ }\left(d,r,n\right)>1-n\left(n-1\right)\cdot \frac{0.5\left(I_{\alpha }\left(\frac{d+1}{2},\frac{1}{2}\right)-{\left(2r\right)}^{d}\cdot I_{\beta }\left(\frac{d+1}{2},\frac{1}{2}\right)\right)}{2^{d}\left(1-r^{d}\right)}.$$

**Proof.**  Denote by $A_{n}$ the event that $M_{n}$ is linearly separable and denote by $C_{i}$ the event that $X_{i}\;\notin \;\mathrm{conv}\left(M_{n}\backslash \left\{X_{i}\right\}\right)$ (i=1,…,n). Thus, $P^{\circ }\left(d,r,n\right)=P\left(A_{n}\right).$ Clearly, $A_{n}=C_{1}\cap \ldots \cap C_{n}$ and $P\left(A_{n}\right)=P\left(C_{1}\cap \ldots \cap C_{n}\right)=1-P\left({\bar{C}}_{1}\cup \ldots \cup {\bar{C}}_{n}\right)\geq 1-\sum_{i=1}^{n}P\left({\bar{C}}_{i}\right).$ Let us find an upper bound for the probability of the event ${\bar{C}}_{i}.$ This event means that the point $X_{i}$ belongs to the convex hull of the remaining points, i.e., $X_{i}\in \mathrm{conv}\left(M_{n}\backslash \left\{X_{i}\right\}\right).$ In the proof of the previous theorem, it was shown that if $0\leq r\leq \frac{1}{\sqrt{2}}$, then

$$P\left({\bar{C}}_{i}\right)\leq \left(n-1\right)\cdot \frac{1-0.5\left(I_{\alpha }\left(\frac{d+1}{2},\frac{1}{2}\right)+{\left(2r\right)}^{d}\cdot I_{\beta }\left(\frac{d+1}{2},\frac{1}{2}\right)\right)}{2^{d}\left(1-r^{d}\right)}\;\left(i=1,\ldots ,n\right);$$

and if $\frac{1}{\sqrt{2}}\leq r<1$, then

$$P\left({\bar{C}}_{i}\right)\leq \left(n-1\right)\cdot \frac{0.5\left(I_{\alpha }\left(\frac{d+1}{2},\frac{1}{2}\right)-{\left(2r\right)}^{d}\cdot I_{\beta }\left(\frac{d+1}{2},\frac{1}{2}\right)\right)}{2^{d}\left(1-r^{d}\right)}\;\left(i=1,\ldots ,n\right).$$

Therefore, using the inequality

$$P\left(A_{n}\right)\geq 1-\sum_{i=1}^{n}P\left({\bar{C}}_{i}\right)$$

we obtain what is required. □

**Figure 6:**
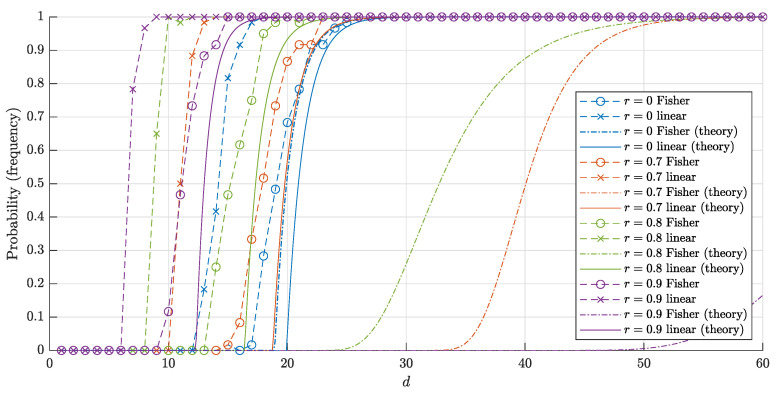
The graphs of the estimates for the probabilities $P^{\circ }\left(d,r,n\right)$ ($P^{\circ F}\left(d,r,n\right)$) that a random set of n=1000 points in $B_{d}\backslash rB_{d}$ is linearly (and respectively Fisher) separable. The solid lines correspond to the theoretical bounds (16) and (17) for the linear separability. The dash-dotted lines represent the theoretical bound (4) and (7) for the Fisher separability. The crosses (circles) correspond to the empirical frequencies for linear (and respectively, Fisher) separability obtained in 60 trials for each dimension *d*.

**Figure 7:**
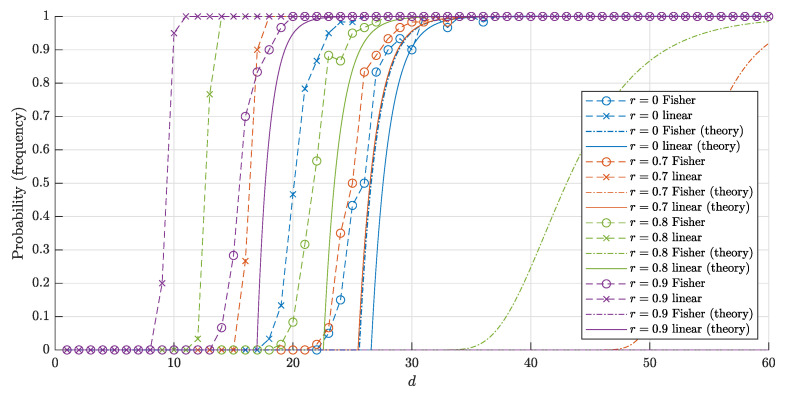
The graphs of the estimates for the probabilities $P^{\circ }\left(d,r,n\right)$ ($P^{\circ F}\left(d,r,n\right)$) that a random set of n=10,000 points in $B_{d}\backslash rB_{d}$ is linearly (and respectively, Fisher) separable. The notation is the same as in Figure 6.

The graphs of the estimates (16), (17) and corresponding frequencies in 60 trials for n=1000 and *n* = 10,000 points are shown in Figure 6 and Figure 7, respectively. The experiment shows that our estimates are quite accurate and close to the corresponding frequencies.

Another important conclusion from the experiment is as follows. Despite the fact that the estimates for both probabilities $P^{\circ F}\left(d,r,n\right)$ and $P^{\circ }\left(d,r,n\right)$ and corresponding frequencies are close to 1 for sufficiently big *d*, the "threshold values" for such a big *d* differ greatly. In other words, the blessing of dimensionality when using linear discriminants comes noticeably earlier than if we only use Fisher discriminants. This is achieved at the cost of constructing the usual linear discriminant in comparison with the Fisher one.

The following corollary gives an estimate for the number of points *n* guaranteeing the linear separability of a random *n*-element set $M_{n}$ in $B_{d}\backslash rB_{d}$ with probability close to 1.

**Corollary** **2.** *Let 0<ϑ<1,$\alpha =4r^{2}\left(1-r^{2}\right),$$\beta =1-r^{2}$. If*

*(1)*  $$0\leq r\leq \frac{1}{\sqrt{2}}\;and\;n<\sqrt{N_{1}\left(d,r,\vartheta \right)}=\sqrt{\frac{\vartheta 2^{d}\left(1-r^{d}\right)}{1-0.5\left(I_{\alpha }\left(\frac{d+1}{2},\frac{1}{2}\right)+{\left(2r\right)}^{d}\cdot I_{\beta }\left(\frac{d+1}{2},\frac{1}{2}\right)\right)}}$$
  *or*
*(2)*  $$\frac{1}{\sqrt{2}}\leq r<1\;and\;n<\sqrt{N_{2}\left(d,r,\vartheta \right)}=\sqrt{\frac{\vartheta 2^{d}\left(1-r^{d}\right)}{0.5\left(I_{\alpha }\left(\frac{d+1}{2},\frac{1}{2}\right)-{\left(2r\right)}^{d}\cdot I_{\beta }\left(\frac{d+1}{2},\frac{1}{2}\right)\right)}},$$
  *then $P^{\circ }\left(d,r,n\right)>1-\vartheta .$*

The theorem below establishes asymptotic estimates for the number of points guaranteeing the linear separability with probability greater than 1−ϑ.

**Theorem** **4.**

*(1)*  *If $0\leq r<\frac{1}{\sqrt{2}}$ then*
  $$\sqrt{N_{1}\left(d,r,\vartheta \right)}∼\sqrt{\vartheta }2^{d/2}.$$
*(2)*  *If $r=\frac{1}{\sqrt{2}}$ then*
  $$\sqrt{N_{1}\left(d,r,\vartheta \right)}=\sqrt{N_{2}\left(d,r,\vartheta \right)}∼\sqrt{\vartheta }2^{(d+1)/2}.$$
*(3)*  *If $\frac{1}{\sqrt{2}}<r<1$ then*
  $$\sqrt{N_{2}\left(d,r,\vartheta \right)}∼\sqrt{\vartheta }\sqrt[{4}]{2\pi }\cdot \frac{\sqrt{r(2r^{2}-1)}}{\sqrt[{4}]{1-r^{2}}}\cdot \sqrt[{4}]{d+1}\cdot {\left(\frac{1}{r\sqrt{1-r^{2}}}\right)}^{d/2}.$$

### 4.3. Comparison of the Results

Let us show that the new estimates (16) and (17) for linear separability tend to be 1 faster than the estimate (4) in [12] for Fisher separability.

**Statement** **1.** *Let 0<r<1,$\alpha =4r^{2}\left(1-r^{2}\right),$$\beta =1-r^{2}$ and d,n∈N,*

$$f_{1}=n\left(n-1\right)\cdot \frac{1-0.5\left(I_{\alpha }\left(\frac{d+1}{2},\frac{1}{2}\right)+{\left(2r\right)}^{d}\cdot I_{\beta }\left(\frac{d+1}{2},\frac{1}{2}\right)\right)}{2^{d}\left(1-r^{d}\right)},$$

$$f_{2}=n\left(n-1\right)\cdot \frac{0.5\left(I_{\alpha }\left(\frac{d+1}{2},\frac{1}{2}\right)-{\left(2r\right)}^{d}\cdot I_{\beta }\left(\frac{d+1}{2},\frac{1}{2}\right)\right)}{2^{d}\left(1-r^{d}\right)},$$

$$g=1-{\left[\left(1-r^{d}\right)\left(1-\left(n-1\right)\frac{{\left(1-r^{2}\right)}^{d/2}}{2}\right)\right]}^{n}.$$

*For r and n fixed*

*(1)*  *if $0<r<\frac{1}{\sqrt{2}}$, then*
  $$\frac{g}{f_{1}}∼\frac{1}{2}{\left(4-4r^{2}\right)}^{d/2}\to \infty ;$$
*(2)*  *if $r=\frac{1}{\sqrt{2}}$, then*
  $$\frac{g}{f_{1}}=\frac{g}{f_{2}}∼\frac{n+1}{n-1}\cdot 2^{d/2}\to \infty ;$$
*(3)*  *if $\frac{1}{\sqrt{2}}<r<1$, then*
  $$\frac{g}{f_{2}}∼\sqrt{2\pi }\cdot \frac{r(2r^{2}-1)}{\left(n-1\right)\sqrt{1-r^{2}}}\cdot \sqrt{d+1}\cdot {\left(\frac{1}{1-r^{2}}\right)}^{d/2}\to \infty .$$

**Proof.**  If $0<r<\frac{1}{\sqrt{2}},$ then $g∼\frac{n(n-1)}{2}{\left(1-r^{2}\right)}^{d/2}$ and $f_{1}∼\frac{n(n-1)}{2^{d}}$ (see the proof of Theorem 2); hence,

$$\frac{g}{f}∼\frac{\frac{n(n-1)}{2}{\left(1-r^{2}\right)}^{d/2}}{\frac{n(n-1)}{2^{d}}}=\frac{1}{2}{\left(4-4r^{2}\right)}^{d/2}\to \infty ,\;\mathrm{as}\;4-4r^{2}>2.$$

If $r=\frac{1}{\sqrt{2}},$ then $g∼\frac{n(n+1)}{2}\frac{1}{2^{d/2}}$ and $f_{1}=f_{2}∼\frac{n(n-1)}{2^{d+1}}$ (see the proof of Theorem 2); hence,

$$\frac{g}{f_{1}}=\frac{g}{f_{1}}∼\frac{\frac{n(n+1)}{2}\frac{1}{2^{d/2}}}{\frac{n(n-1)}{2^{d+1}}}=\frac{n+1}{n-1}\cdot 2^{d/2}\to \infty .$$

If $\frac{1}{\sqrt{2}}<r<1,$ then $g∼nr^{d}$ and $f_{2}∼\frac{n(n-1)}{\sqrt{2\pi }\cdot \frac{r(2r^{2}-1)}{\sqrt{1-r^{2}}}\cdot \sqrt{d+1}\cdot {\left(\frac{1}{r\sqrt{1-r^{2}}}\right)}^{d}}$ (see the proof of Theorem 2), hence

$$\frac{g}{f_{2}}∼\frac{nr^{d}\sqrt{2\pi }\cdot \frac{r(2r^{2}-1)}{\sqrt{1-r^{2}}}\cdot \sqrt{d+1}\cdot {\left(\frac{1}{r\sqrt{1-r^{2}}}\right)}^{d}}{n(n-1)}=\sqrt{2\pi }\cdot \frac{r(2r^{2}-1)}{\left(n-1\right)\sqrt{1-r^{2}}}\cdot \sqrt{d+1}\cdot {\left(\frac{1}{1-r^{2}}\right)}^{d/2}\to \infty .$$

 □

Now let us compare the estimates for the number of points that guarantee the linear and Fisher separabilities of random points in the spherical layer obtained in Corollary 2 and in [12], respectively. The estimate in Corollary 2 for the number of points guaranteeing the linear separability tends to ∞ faster than the estimate (5), guaranteeing the Fisher separability for all 0<r<1.

**Statement** **2.** *Let $f_{1}=\sqrt{N_{1}\left(d,r,\vartheta \right)},$$f_{2}=\sqrt{N_{2}\left(d,r,\vartheta \right)},$$g=\frac{\sqrt{\vartheta }}{{\left(1-r^{2}\right)}^{d/4}},$0<r<1,0<ϑ<1,d∈N. For r and ϑ fixed*

*(1)*  *if $0<r<\frac{1}{\sqrt{2}}$, then*
  $$\frac{f_{1}}{g}∼{\left(2\sqrt{1-r^{2}}\right)}^{d/2}\to \infty ;$$
*(2)*  *if $r=\frac{1}{\sqrt{2}}$, then*
  $$\frac{f_{1}}{g}=\frac{f_{2}}{g}∼2^{(d+2)/4}\to \infty ;$$
*(3)*  *if $\frac{1}{\sqrt{2}}<r<1$, then $\frac{f_{2}}{g}∼\sqrt{\sqrt{2\pi }\cdot \frac{r(2r^{2}-1)}{\sqrt{1-r^{2}}}}\cdot {\left(d+1\right)}^{1/4}\cdot {\left(\frac{1}{r}\right)}^{d/2}\to \infty .$*

**Proof.**  If $0<r<\frac{1}{\sqrt{2}}$ then $\frac{f_{1}}{g}∼\frac{\sqrt{\vartheta 2^{d}}{\left(1-r^{2}\right)}^{d/4}}{\sqrt{\vartheta }}={\left(2\sqrt{1-r^{2}}\right)}^{d/2}.$

If $r=\frac{1}{\sqrt{2}}$, then $f_{1}=f_{2}∼\sqrt{\vartheta 2^{d+1}}$ and $g=\sqrt{\vartheta }2^{d/4}$; hence, $\frac{f_{1}}{g}=\frac{f_{2}}{g}∼\frac{\sqrt{\vartheta 2^{d+1}}}{\sqrt{\vartheta }2^{d/4}}=2^{(d+2)/4}.$

If $\frac{1}{\sqrt{2}}<r<1$, then $f_{2}∼\sqrt{\vartheta \sqrt{2\pi }\cdot \frac{r(2r^{2}-1)}{\sqrt{1-r^{2}}}\cdot \sqrt{d+1}\cdot {\left(\frac{1}{r\sqrt{1-r^{2}}}\right)}^{d}}$; hence,

$$\frac{f_{2}}{g}∼\sqrt{\vartheta \sqrt{2\pi }\cdot \frac{r(2r^{2}-1)}{\sqrt{1-r^{2}}}}\cdot {\left(d+1\right)}^{1/4}\cdot {\left(\frac{1}{r^{2}\left(1-r^{2}\right)}\right)}^{d/4}\frac{{\left(1-r^{2}\right)}^{d/4}}{\sqrt{\vartheta }}$$

$$=\sqrt{\sqrt{2\pi }\cdot \frac{r(2r^{2}-1)}{\sqrt{1-r^{2}}}}\cdot {\left(d+1\right)}^{1/4}\cdot {\left(\frac{1}{r}\right)}^{d/2}.$$

 □

### 4.4. A Note about Random Points Inside the Ball (r=0)

A reviewer of the original version of the article drew our attention to the fact that for the uniform distribution inside the ball (case r=0), better results are known. Specifically, let ${\bar{p}}_{xy}^{F}$ be the probability that i.i.d. points *x*, *y* inside the ball are not Fisher separable. Let $I_{xy}$ be the indicator function of this event. Then

$${\bar{p}}_{xy}^{F}=E\left[I_{xy}\right]=E\left[E\left[I_{xy}|y\right]\right]=E\left[{\bar{p}}_{y}\right],$$

where ${\bar{p}}_{y}$ denotes the probability that *x* is not Fisher separable from a given point *y*. In [6] (also discussed in [15]), there is a proof that $E\left[{\bar{p}}_{y}\right]=1/2^{d+1}$. In the notation of our paper, this implies that

$$P_{1}^{\circ F}\left(d,0,n\right)\geq 1-\frac{n}{2^{d+1}},\;P^{\circ F}\left(d,0,n\right)\geq 1-\frac{n(n-1)}{2^{d+1}}>1-\frac{n^{2}}{2^{d+1}},$$

and $P_{1}^{\circ F}\left(d,0,n\right)>1-\vartheta$ provided that $n<\vartheta \cdot 2^{d+1}$. This improves the estimate in Theorem 2 for the case r=0 twice. Note that the same estimate $n<\vartheta \cdot 2^{d+1}$ was derived for $r=\frac{1}{\sqrt{2}}$ (see Theorem 2). The reviewer conjectured that estimate $n<\vartheta \cdot 2^{d}$ derived in this paper could be improved twice for the whole range $r\in \left[0,\frac{1}{\sqrt{2}}\right)$. The experimental results give support for this hypothesis (see Figure 4, Figure 5, Figure 6 and Figure 7).

## 5. Random Points Inside a Cube

Consider a set of points $M_{n}=\left\{X_{1},\ldots ,X_{n}\right\}$ choosing randomly, independently and according to the uniform distribution on the *d*-dimensional unit cube $Q_{d}$.

**Theorem** **5.** *Let d,n∈N. Then*

(18) $$P_{1}^{\;□}\left(d,n\right)>1-\frac{n(d+1)}{c^{d}},\;c=1.18858\ldots$$

**Proof.**  A random point *Y* is linearly separable from $M_{n}=\left\{X_{1},\ldots ,X_{n}\right\}$ if and only if $Y\notin \mathrm{conv}(M_{n}).$ Denote this event by C. Thus, $P_{1}^{\;□}\left(d,n\right)=P\left(C\right).$ Let us find the upper bound for the probability of the event $\bar{C}.$ This event means that the point *Y* belongs to the convex hull of $M_{n}.$ Since the points in $M_{n}$ have the uniform distribution, the probability of $\bar{C}$ is

$$P\left(\bar{C}\right)=\frac{\mathrm{Vol}\left(\mathrm{conv}(M_{n})\right)}{\mathrm{Vol}(Q_{d})}=\mathrm{Vol}\left(\mathrm{conv}(M_{n})\right).$$

In [20] it is proved that the upper bound for the maximal volume of the convex hull of *k* points placed in $Q_{d}$ is $\frac{k(d+1)}{c^{d}},$ where c=1.18858. Thus, $\mathrm{Vol}\left(\mathrm{conv}(Y_{1},\ldots ,Y_{k})\right)<\frac{k(d+1)}{c^{d}}$ so

$$P\left(\bar{C}\right)=\mathrm{Vol}\left(\mathrm{conv}(M_{n})\right)\;<\frac{n(d+1)}{c^{d}}.$$

and

$$P_{1}^{\;□}\left(d,n\right)=P\left(C\right)=1-P\left(\bar{C}\right)>1-\frac{n(d+1)}{c^{d}}.$$

 □

**Corollary** **3.** *Let 0<ϑ<1,*

(19) $$n<\frac{\vartheta c^{d}}{d+1},\;c=1.18858\ldots$$

*Then $P_{1}^{\;□}\left(d,n\right)>1-\vartheta .$*

**Theorem** **6.** *Let d,n∈N. Then*

(20) $$P^{\;□}\left(d,n\right)>1-\frac{n(n-1)(d+1)}{c^{d}},\;c=1.18858.$$

**Proof.**  Denote by $A_{n}$ the event that $M_{n}$ is linearly separable and denote by $C_{i}$ the event that $X_{i}\;\notin \;\mathrm{conv}\left(M_{n}\backslash \left\{X_{i}\right\}\right)$ (i=1,…,n). Thus, $P^{□}\left(d,n\right)=P\left(A_{n}\right).$ Clearly $A_{n}=C_{1}\cap \ldots \cap C_{n}$ and $P\left(A_{n}\right)=P\left(C_{1}\cap \ldots \cap C_{n}\right)=1-P\left({\bar{C}}_{1}\cup \ldots \cup {\bar{C}}_{n}\right)\geq 1-\sum_{i=1}^{n}P\left({\bar{C}}_{i}\right).$ Let us find the upper bound for the probability of the event ${\bar{C}}_{i}.$ This event means that the point $X_{i}$ belongs to the convex hull of the remaining points, i.e., $X_{i}\in \mathrm{conv}\left(M_{n}\backslash \left\{X_{i}\right\}\right).$ In the proof of the previous theorem, it was shown that

$$P\left({\bar{C}}_{i}\right)\leq \frac{\left(n-1)(d+1\right)}{c^{d}},\;c=1.18858\;\left(i=1,\ldots ,n\right).$$

Hence

$$P\left(A_{n}\right)\geq 1-\sum_{i=1}^{n}P\left({\bar{C}}_{i}\right)\geq 1-\frac{n(n-1)(d+1)}{c^{d}}.$$

 □

**Corollary** **4.** *[13] Let 0<ϑ<1,*

(21) $$n<\sqrt{\frac{\vartheta c^{d}}{d+1}},\;c=1.18858.$$

*Then $P^{\;□}\left(d,n\right)>1-\vartheta .$*

We note that the estimate (21) for the number of points guaranteeing the linear separability tends to be ∞ faster than the estimate (13), guaranteeing the Fisher separability because

$$\frac{\sqrt{\frac{\vartheta c^{d}}{d+1}}}{\sqrt{\frac{\vartheta e^{d/288}}{3}}}=\sqrt{\frac{3}{d+1}{\left(\frac{c}{e^{\frac{1}{288}}}\right)}^{d}}\to \infty ,\;\mathrm{as}\;d\to \infty ,$$

since $c/e^{\frac{1}{288}}\approx 1.18446.$

However better (and in fact asymptotically optimal) estimates for the Fisher separability in the unit cube are derived in [15]. Corollary 7 in [15] states that *n* points are Fisher separable with probability greater than 1−ϑ provided only that $n<\sqrt{\vartheta }e^{\gamma d}$ for γ=0.23319…. This can be written as $n<\sqrt{\vartheta c^{d}}$ for $c=e^{2\gamma }=1.59421\ldots$. Thus,

(22) $$P_{1}^{\;□F}\left(d,n\right)>1-\frac{n}{\exp(2\gamma d)}=1-\frac{n}{c^{d}},$$

(23) $$P^{\;□F}\left(d,n\right)>1-\frac{n^{2}}{c^{d}}.$$

Theorem 6 and Corollary 4 in our paper state the same results with c=1.18858…, and for just linear separability instead of Fisher separability. However, [13,15] were submitted to the same conference, so these results were derived in parallel and independently.

**Figure 8:**
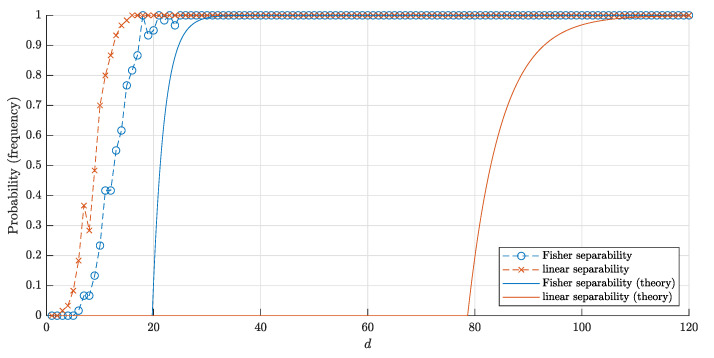
The graphs of the estimate for the probabilities $P_{1}^{\;□}\left(d,n\right)$ and $P_{1}^{\;□\;F}\left(d,n\right)$ that a random point is linearly (Fisher) separable from a set of *n* = 10,000 random points inside the cube $Q_{d}$. The solid red and blue lines correspond to the theoretical bounds (18) and (22) respectively. Red crosses (blue circles) correspond to the empirical frequencies for linear (and respectively, Fisher) separability obtained in 60 trials for each dimension *d*.

**Figure 9:**
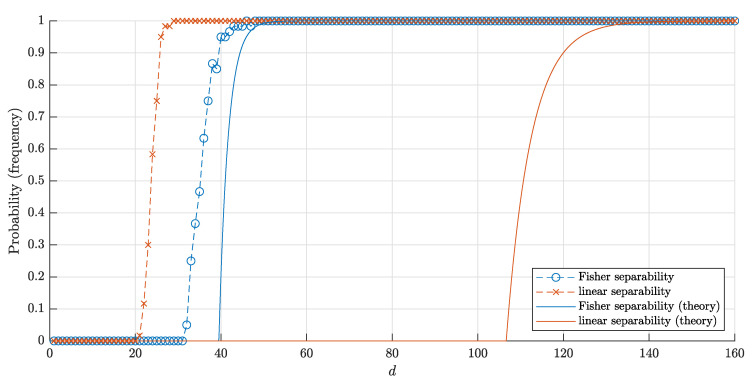
The graphs of the estimates (20) and (23) for the probabilities $P^{\;□}\left(d,n\right)$ and $P^{\;□\;F}\left(d,n\right)$ that a set of *n* = 10,000 random points inside the unit cube $Q_{d}$ is linear and Fisher separable, respectively. The notation is the same as in Figure 8.

The bounds (18) and (20) for the probabilities and corresponding frequencies are presented in Figure 8 and Figure 9.

## 6. Subsequent Work

In a recent paper [2], explicit and asymptotically optimal estimates of Fisher separation probabilities for spherically invariant distribution (e.g., the standard normal and the uniform distributions) were obtained. Theorem 14 in [2] generalizes the results presented here. Since [2] was submitted to the arxiv later, we did not compare the results of that article with our results.

## 7. Conclusions

In this paper we refined the estimates for the number of points and for the probability in stochastic separation theorems. We gave new bounds for linear separability, when the points are drawn randomly, independently and uniformly from a *d*-dimensional spherical layer or from the unit cube. These results refine some results obtained in [5,12,13,14] and allow us to better understand the applicability limits of the stochastic separation theorems for high-dimensional data mining and machine learning problems.

The strongest progress was in the estimation for the number of random points in a (1−r)-thick spherical layer $B_{d}\backslash rB_{d}$ that are linear separable with high probability. If

$$n≲\sqrt{\vartheta }2^{d/2},\;0\leq r<\frac{1}{\sqrt{2}}\;\mathrm{or}\;n≲\sqrt{\vartheta }2^{(d+1)/2},\;r=\frac{1}{\sqrt{2}}$$

or

$$n≲\sqrt{\vartheta }\sqrt[{4}]{2\pi }\cdot \frac{\sqrt{r(2r^{2}-1)}}{\sqrt[{4}]{1-r^{2}}}\cdot \sqrt[{4}]{d+1}\cdot {\left(\frac{1}{r\sqrt{1-r^{2}}}\right)}^{d/2},\;\frac{1}{\sqrt{2}}<r<1,$$

then *n* i.i.d. random points inside the spherical layer $B_{d}\backslash rB_{d}$ are linear separable with probability at least 1−ϑ (the asymptotic inequalities are for d→∞).

One of the main results of the experiment comparing linear and Fisher separabilities is as follows. The blessing of dimensionality when using linear discriminants can come noticeably earlier (for smaller values of *d*) than if we only use Fisher discriminants. This is achieved at the cost of constructing the usual linear discriminant in comparison with the Fisher one.
